# Scoliosis in Shprintzen–Goldberg Syndrome

**DOI:** 10.1155/2020/8857463

**Published:** 2020-11-23

**Authors:** Hiromitsu Takano, Ikuho Yonezawa, Takatoshi Okuda, Hajime Kajihara, Kazuo Kaneko

**Affiliations:** ^1^Department of Orthopedic Surgery, Koto Hospital, 6-8-5 Ojima, Koto-ku, Tokyo 136-0072, Japan; ^2^Department of Orthopedic Surgery, Juntendo University School of Medicine, 2-1-1 Hongo, Bunkyo-ku, Tokyo 113-8421, Japan; ^3^Department of Spine Surgery, Sangubashi Spine Surgery Hospital, 3-57-1 Yoyogi, Shibuya-ku, Tokyo 151-0053, Japan

## Abstract

We report a case of scoliosis in a 12-year-old girl with Shprintzen–Goldberg syndrome. She was diagnosed with Shprintzen–Goldberg syndrome at birth. She was hospitalized for a surgical treatment because scoliosis gradually progressed. Preoperative X-ray confirmed 80° symptomatic scoliosis in T10–L5. Posterior correction and fusion were performed, and postoperative X-ray showed a correction to 43°in T10-L5. Limited subcutaneous tissues and fragile bones must be considered when selecting the appropriate surgical method. Accurate placement of a screw into thin pedicle is essential to obtain sufficient correction and fusion. The use of a navigation system is recommended.

## 1. Introduction

Shprintzen–Goldberg syndrome is similar to Marfan syndrome, which presents diverse symptoms of systemic weakness in the connective tissues in the skeletal system, cardiovascular system, and eyes. We treated a case of scoliosis in a patient with Shprintzen–Goldberg syndrome and reported the results.

## 2. Case Presentation

This study reports a case of a 12-year-old girl (height, 138 cm; weight, 26 kg) with a chief complaint of scoliosis; a medical history of craniosynostosis and bilateral habitual dislocation of patella; and no family history of the same illness.

The subject was diagnosed with Shprintzen–Goldberg syndrome at birth and was followed up at our pediatrics department. The patient visited our department at the age of four years and was diagnosed with symptomatic levoscoliosis (T12–L5: 56° Lenke 5c), and brace therapy was started. The patient was hospitalized for a surgical procedure at the age of 12 years because deformation progressed gradually.

**Figure 1:**
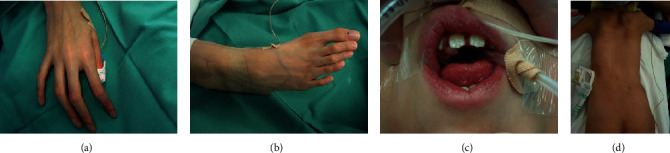
(a, b) Arachnodactyly and contractures of the proximal interphalangeal joints, (c) dental malpositioning, and (d) emaciation and minimal subcutaneous tissues.

Delayed mental development, ligamentous laxity, emaciation, arachnodactyly, and dental malpositioning were confirmed (Figure 1). No neurological abnormality was found.

**Figure 2:**
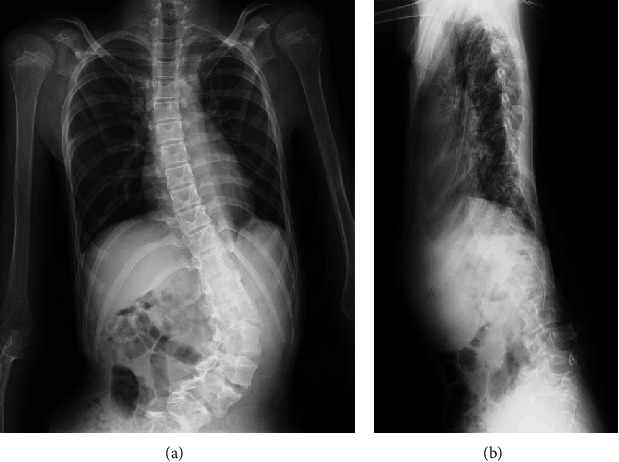
(a) Preoperative X-ray—anterior-posterior (AP) view of levoscoliosis (T10–L5: 80°) and (b) preoperative X-ray—lateral view.

**Figure 3:**
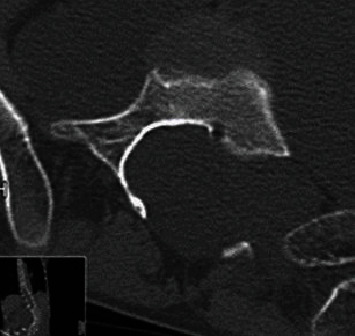
Preoperative axial CT. Dural ectasia and massive intraperitoneal myelomeningocele.

Preoperative X-ray confirmed levoscoliosis (T10–L5: 80°) (Figure 2), and computed tomography (CT) scan confirmed dural ectasia and massive intraperitoneal myelomeningocele (Figure 3).

**Figure 4:**
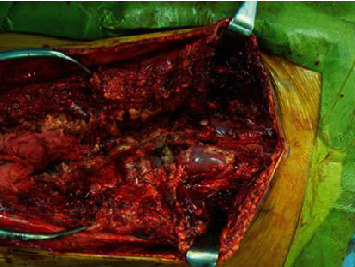
Intraoperative photo; myelomeningocele was confirmed at the posterior left side L5 level.

**Figure 5:**
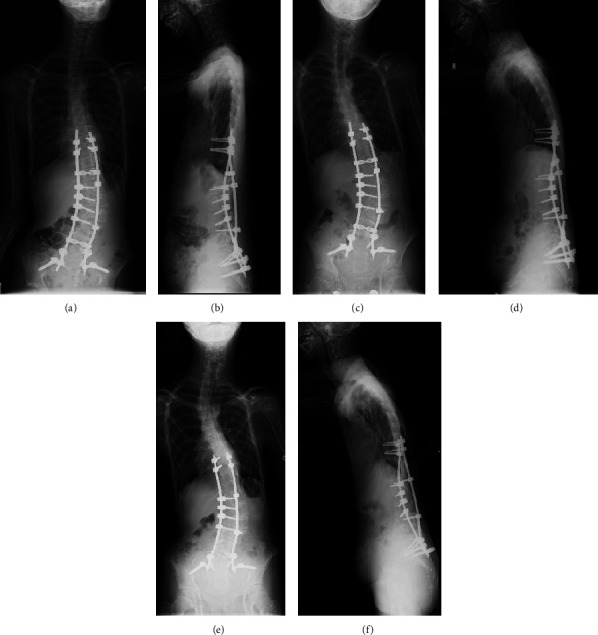
(a) Postoperative X-ray—AP view, levoscoliosis (T10–L5: 43°); (b) postoperative X-ray—lateral view; (c) rod breakage X-ray—AP view; (d) rod breakage X-ray—lateral view; (e) last follow-up X-ray—AP view; (f) last follow-up X-ray—lateral view.

**Figure 6:**
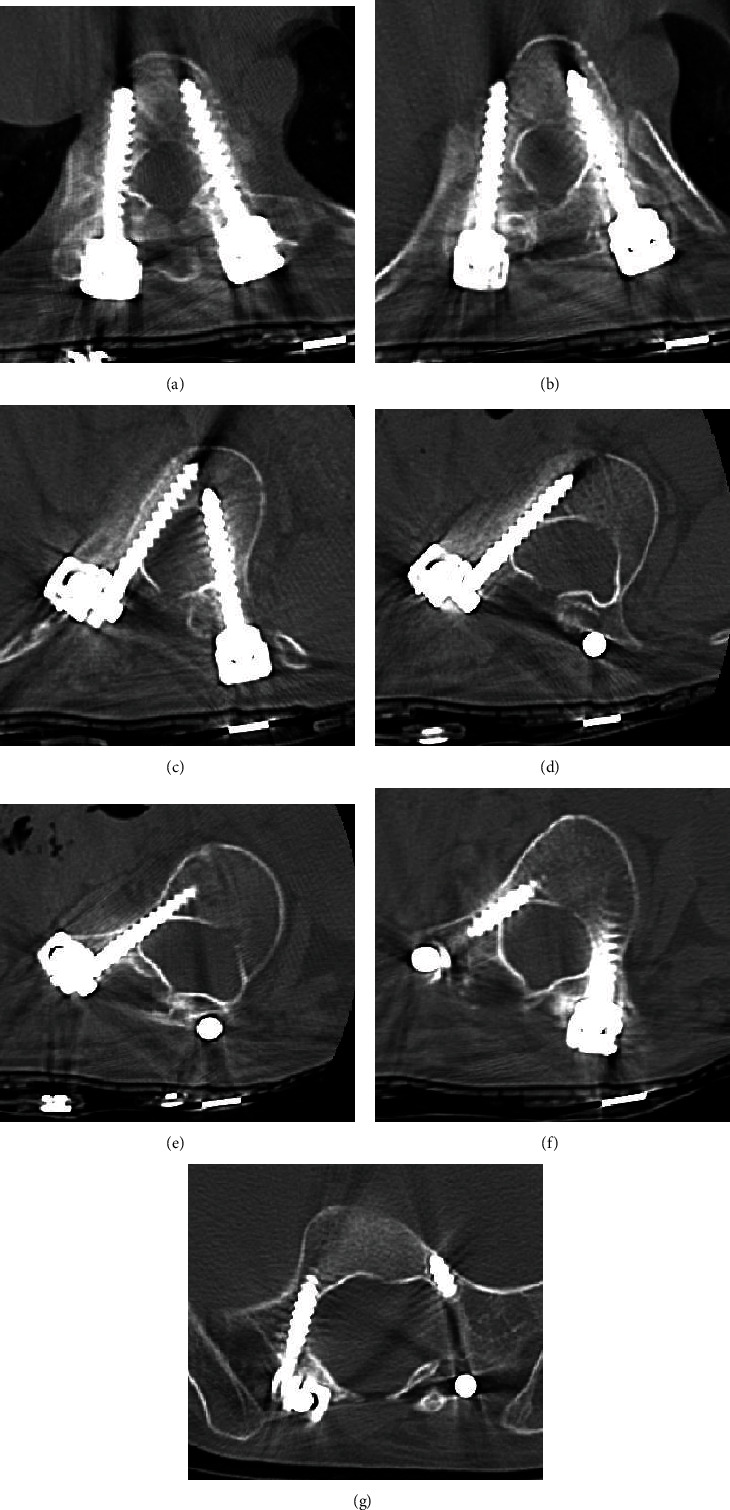
Postoperative axial CT: (a) T9, (b) T10, (c) L1, (d) L2, (e) L3, (f) L4, and (g) S1.

Using the navigation system (Medtronic® StealthStation®), pedicle and iliac screws were inserted bilaterally into T9, T10, L1, L4, and S1, as well as the right side of L2 and L3. Posterior correction and fusion were performed during MEP and SEP monitoring. Myelomeningocele was confirmed during the surgery (Figure 4). Postoperative correction was performed at T10–L5: 43° (Figures 5(a) and 5(b)). A break in a rod was confirmed one year and three months postoperatively (Figures 5(c) and 5(d)); and thus, the rod was replaced and fused again. No correction loss was observed nine years postoperatively. The vertebra has autofused, and follow-up results are favorable (Figures 5(e) and 5(f)). The pedicle was extremely thin, and for many pedicles, free-hand insertion of pedicle screws was considered difficult. Therefore, accurate insertion of pedicle screws by using a navigation system was considered essential in obtaining sufficient correction and fusion (Figure 6).

## 3. Discussion

Shprintzen–Goldberg syndrome is similar to Marfan syndrome, causing diverse symptoms due to systemic weakness in the connective tissues in the skeletal system, cardiovascular system, and eyes [1, 2]. TGFBR2 missense mutation has been identified as the causative gene [3–7], and typical clinical symptoms include cardiac malformation, arachnodactyly, and craniosynostosis. Characteristics of scoliosis are progressive and severe spinal deformities such as fragile bones, pedicle with dural ectasia, and hypoplasia of lamina [8–11]. Approximately 30% of cases are reported to have craniocervical deformation, and Jödicke et al. reported the use of a surgical treatment (occipitocervical fusion) for craniocervical instability associated with C1 deformation [12]. In the surgical treatment of spinal deformation, a posterior approach is often selected because of weakness in the abdominal wall and great vessels. It should also be noted that because minimal subcutaneous tissues are present, low-profile internal instrumentation should be selected. Normally, if it progresses quickly during infancy, the use of a growing rod may be considered; however, Watanabe et al. reported that correction with this disease using growing rods in one out of two cases of spinal deformity required removal of internal instrumentation because of infection, whereas an incident of infection and three dislodgements were found in another case [11]. Considering the weakness in the skin tissue that is characteristic of this disease, multiple surgeries for the growing rod method are considered high risk. Considering that it is flexible, spinal deformation in this disease should be treated with a brace as long as possible, and a single surgery for posterior correction should be performed. We waited until the Cobb angle reached 80° and performed a single posterior fusion, which resulted in a sufficient correction. In this case, myelomeningocele was also found, and there was a concern that posterior fusion would be difficult. However, despite one break in the rod, a replacement of the rod was required. Ultimately, the vertebral body naturally healed, leading to a favorable result. The biggest issue with the posterior correction and fusion of this disease is the hypoplasia of the pedicle. In the present case, the pedicle was extremely thin (at the thinnest pedicle diameter 3 mm), and for many pedicles, free-hand insertion of pedicle screws was considered difficult. Therefore, accurate insertion of pedicle screws by using a navigation system was considered essential in obtaining sufficient correction and fusion.

## 4. Conclusions

We reported our treatment of a case of scoliosis in a patient with Shprintzen–Goldberg syndrome. A surgical method must be selected with consideration given to the weakness of the skin and bones. Accurate placement of the screw in the thin pedicle is essential to obtain sufficient correction and fusion, and thus, the use of a navigation system is recommended.
